# Epidemiological and clinical characteristics of children with confirmed COVID-19 infection in a tertiary referral hospital in Manila, Philippines

**DOI:** 10.1186/s41182-023-00507-x

**Published:** 2023-02-22

**Authors:** Rhanee Lota-Salvado, Jay Ron Padua, Kristal An Agrupis, Greco Mark Malijan, Ana Ria Sayo, Shuichi Suzuki, Grace Devota Go, Chris Smith

**Affiliations:** 1 School of Tropical Medicine and Global Health, Nagasaki University, Nagasaki, Japan; 2 San Lazaro Hospital, Manila, Philippines; 3 San Lazaro Hospital–Nagasaki University Collaborative Research Office, Manila, Philippines; 4 Department of Clinical Research, London School of Hygiene and Tropical Medicine, London, UK

**Keywords:** COVID-19, Epidemiology, Philippines, Children, Pediatric, Low- and middle-income countries (LMIC)

## Abstract

**Background:**

COVID-19 has challenged the under-resourced health systems of low- and middle-income countries, significantly affecting child health. Available published data on Filipino children with COVID-19 infection are limited. This study aims to describe the epidemiological and clinical characteristics of pediatric patients with confirmed COVID-19 in an infectious disease hospital in Manila, Philippines.

**Main text:**

This cross-sectional study reviewed data on patients ages 0 to 18 years with confirmed COVID-19 infection, admitted to San Lazaro Hospital from January 25, 2020 to January 25, 2022. Demographic data and clinical characteristics obtained from COVID-19 case investigation forms were summarized and compared between severe and non-severe cases. Risk factors for disease severity and mortality were analyzed. Of 115 patients, 64% were males. There were 87 patients (75.7%) with asymptomatic, mild, or moderate disease, and 28 cases (24.3%) with severe or critical illness. The median age of all patients was 10 years (interquartile range: 4–15 years). The majority of patients (40.9%) were adolescents ages 13 to 18 years. Predominant symptoms were fever (73.9%) and cough (55.7%). Patients with severe or critical illness were more likely to experience difficulty of breathing (55.2% vs 44.8%, *p* < 0.001), and have a longer hospital stay (11 days vs 8 days, *p* = 0.043). Among all patients, 48.7% had at least one underlying disease; and common infectious co-morbidities were tuberculosis (17.4%), dengue (12.2%), and HIV (4.3%). Having tuberculosis (*p* = 0.008) or at least one co-morbidity (*p* < 0.001) was associated with disease severity. Ten patients (8.7%) died; and mortality was higher among those with severe or critical illness (80% vs 20%, *p* < 0.001). Sepsis (*p* = 0.020) or having at least one co-morbidity (*p* = 0.007) was associated with death.

**Conclusion:**

Children of all ages remain susceptible to COVID-19 infection, and usually present with mild or moderate symptoms. In this study, many adolescents are affected, highlighting the value of COVID-19 vaccination in this age group. Understanding the clinical features of COVID-19 in Filipino children is essential to identifying and optimally managing those at highest risk of severe disease.

## Background

COVID-19 infections, caused by the emerging pathogen SARS-CoV-2, have been reported in all age groups. Children of all ages are affected, though to a lesser extent than adults, comprising a small proportion of the total number of reported COVID-19 cases during the early months of the pandemic [1–3]. Since then, reported pediatric infections have increased in number [4]. Physiological distinctions between children and adults have been proposed to explain the difference in severity of COVID-19 infections, including variations in ACE2 expression, immune responses, and endothelial or clotting functions [5–7]. Aggregate global data on pediatric COVID-19 cases and deaths are limited, making it difficult to adequately assess the impact of the disease in the lives of children [8]. Children of all ages remain at risk of developing the disease and the effects of COVID-19 on their lives may be profound and enduring [9, 10].

COVID-19 has challenged the under-resourced health systems of low- and middle-income countries (LMICs). The indirect effects of the pandemic on child health are significant, including reduced access to health services, disrupted schooling, and increasing poverty levels [9–11].

Studies on pediatric COVID-19 in LMICs in the Southeast Asian region are limited. In the Philippines, epidemiologic and clinical data studies on COVID-19 infection involved mostly adult patients [12–14]. To date, there are few available published data on Filipino children with COVID-19 infection. This study aims to add to this knowledge gap by describing the epidemiological and clinical characteristics of pediatric patients with confirmed COVID-19 in an infectious disease hospital in Manila, Philippines.

## Main text

This was a cross-sectional study of patients ages 0 to 18 years with COVID-19 infection confirmed by SARS-CoV-2 RT-PCR testing, admitted to San Lazaro Hospital (SLH) over a 2-year period from January 25, 2020 to January 25, 2022. San Lazaro Hospital is the national infectious disease referral hospital in Manila, Philippines. At the height of the COVID-19 pandemic, pediatric patients were allocated to two wards: a non-COVID ward with a maximum of 52 beds, and a COVID ward which had 40 beds. These patients were managed by the healthcare staff of the Pediatric Infectious Diseases and Tropical Medicine Department. Anonymized data from COVID-19 Case Investigation Forms (CIFs) were provided by the SLH Epidemiology Department (SLH ED). CIFs are forms used by the Philippines Department of Health (DOH) for disease surveillance and monitoring, and completed for all suspected COVID-19 cases.

All data were analyzed using descriptive statistics. Only confirmed cases were included in this study, defined in local guidelines as “any individual, irrespective of presence or absence of clinical signs and symptoms, who was laboratory confirmed for COVID-19 in a test conducted at the national reference laboratory, a subnational reference laboratory, and/or DOH-licensed COVID-19 testing laboratory” [15]. Classification of COVID-19 disease severity in children, as mild, moderate, severe, or critical disease, was done according to DOH and WHO guidelines [16, 17]. Basic demographic characteristics and clinical profiles of the study population were summarized by age, sex, symptoms, co-morbidities, vaccination history, interval between onset of symptoms and admission, duration of hospitalization, clinical status, and outcome. Co-morbidity was identified as “one or more additional diseases occurring concomitantly with a primary disease or disorder” [18]. Analyses were performed on risk factors for severe disease and death. Continuous variables were expressed as mean (standard deviation, SD) and median (interquartile range, IQR) while categorical variables were expressed as frequency (%). Stata 17 was used for all analyses, utilizing Fisher’s exact test and Mann–Whitney U test.

Analysis of the epidemiologic and clinical features of 115 pediatric COVID-19 admissions to SLH is presented in Table 1. Patients were categorized into those who had asymptomatic, mild or moderate disease (*n* = 87); and those who had severe or critical disease (*n* = 28). The first child with confirmed COVID-19 infection was admitted on May 4, 2022.

**Table 1:** Epidemiologic and clinical features of children with COVID-19 admitted to San Lazaro Hospital, January 25, 2020 to January 25, 2022 (*n* = 115)

Characteristics	Total, *n* = 115	Asymptomatic/mild/moderate disease, *n* = 87	Severe/critical disease, *n* = 28	*p*-value^a^
Age (years)				
Mean (SD)	9.5 (6.1)	9.3 (5.9)	10.1 (6.7)	0.449
Median (IQR)	10 (4, 15)	9 (4, 15)	10.5 (5, 17)	
Age group, *n* (%)				
< 1 year old	14 (12.2)	8 (57.1)	6 (42.9)	0.180
1–5 years old	20 (17.4)	18 (90.0)	2 (10)	
6–12 years old	34 (29.5)	26 (76.5)	8 (23.5)	
13–18 years old	47 (40.9)	35 (74.5)	12 (25.5)	
Sex, *n* (%)				
Female	42 (36.5)	35 (83.3)	7 (16.7)	0.179
Male	73 (63.5)	52 (71.2)	21 (28.8)	
Reported symptoms, *n* (%)				
No symptoms	1 (0.9)	1 (100.0)	0 (0.0)	1.000
Fever	85 (73.9)	66 (77.7)	19 (22.3)	0.460
Respiratory				
Cough	64 (55.7)	47 (73.4)	17 (26.6)	0.663
Coryza	25 (21.7)	23 (92.0)	2 (8.0)	0.035
Sore throat	13 (11.3)	8 (61.5)	5 (38.5)	0.300
Difficulty of breathing	29 (25.2)	13 (44.8)	16 (55.2)	< 0.001
Gastrointestinal				
Diarrhea	11 (9.6)	8 (72.7)	3 (27.3)	0.728
Loss of appetite	16 (13.9)	11 (68.8)	5 (31.2)	0.534
Vomiting	22 (19.1)	18 (81.8)	4 (18.2)	0.586
Abdominal pain	12 (10.4)	10 (83.3)	2 (16.7)	0.728
Neurologic				
Headache	11 (9.6)	10 (90.9)	1 (9.1)	0.290
Seizures	4 (3.5)	3 (75.0)	1 (25.0)	1.000
Others				
Anosmia	4 (3.5)	4 (100.0)	0 (0.0)	0.571
Ageusia	6 (5.2)	5 (83.3)	1 (16.7)	1.000
Body weakness	12 (10.4)	10 (83.3)	2 (16.7)	0.728
Rashes	5 (4.3)	3 (60.0)	2 (40.0)	0.594
Interval between onset of symptoms and admission (days)				
Mean (SD)	11 (22.3)	11.6 (23.0)	12.2 (20.3)	0.015
Median (IQR)	5 (3, 8)	5 (3, 7)	6.5 (4, 12.5)	
0–7 days	83 (72.2)	68 (81.9)	15 (18.1)	
8–14 days	18 (15.7)	9 (50.0)	9 (50.0)	
> 15 days	14 (12.2)	10 (71.4)	4 (28.6)	
Duration of hospitalization (days)				
Mean (SD)	11 (11.4)	9.6 (7.4)	18.5 (17.8)	0.043
Median (IQR)	8 (6, 12)	8 (5, 11)	11 (6, 24.5)	
Co-morbidity^b^, *n* (%)				
Tuberculosis, any form	20 (17.4)	10 (50.0)	10 (50.0)	0.008
Dengue	14 (12.2)	12 (85.7)	2 (14.3)	0.512
HIV	5 (4.3)	2 (40.0)	3 (60.0)	0.092
Other illnesses aside from TB, dengue, HIV	21 (18.3)	10 (47.6)	11 (52.4)	0.003
≥ 1 underlying disease	56 (48.7)	32 (57.1)	24 (42.9)	< 0.001
COVID-related diagnosis, *n* (%)				
Pneumonia	44 (38.3)	29 (65.9)	15 (34.1)	0.074
Acute upper respiratory infection	22 (19.1)	21 (95.5)	1 (4.5)	0.014
Acute respiratory distress syndrome	2 (1.7)	0 (0.0)	2 (100.0)	0.058
Acute gastroenteritis	5 (4.3)	5 (100.0)	0 (0.0)	0.333
Multisystem inflammatory syndrome in children (MIS-C)	2 (1.7)	0 (0.0)	2 (100.0)	0.058
Sepsis	3 (2.6)	1 (33.3)	2 (66.7)	0.146
Myocarditis	1 (0.9)	1 (100.0)	0 (0.0)	1.000
Neurologic^c^	4 (3.5)	3 (75.0)	1 (25.0)	1.000
Vaccination status, *n* (%)				
Not vaccinated	113 (98.3)	85 (75.2)	28 (24.8)	1.000
Vaccinated	2 (1.7)	2 (100.0)	0 (0.0)	
Outcome, *n* (%)				
Died	10 (8.7)	2 (20.0)	8 (80.0)	< 0.001
Discharged	105 (91.3)	85 (80.9)	20 (19.1)	

^a^*p*-value from Fisher’s exact test (categorical variables) or Mann–Whitney U test (continuous variables). A *p*-value less than 0.05 was considered statistically significant

^b^Co-morbidity: one or more additional diseases occurring concomitantly with a primary disease or disorder [18]

^c^Neurologic: seizure disorder, encephalitis

The median age of all patients was 10 years (IQR 4–15 years). Many patients were adolescents ages 13 to 18 years (40.9%). Almost two thirds of the patients were male (63.5%). Predominant symptoms were fever (73.9%) and respiratory symptoms such as cough (55.7%), difficulty of breathing (25.2%), coryza (21.7%), and sore throat (11.3%). Common gastrointestinal symptoms were vomiting (19.1%) and loss of appetite (13.9%). Less common symptoms were ageusia (5.2%), rashes (4.3%), anosmia (3.5%), and seizures (3.5%). One patient was asymptomatic. Children with mild or moderate illness were more likely to have coryza than those with severe or critical disease (92% vs 8%, *p* = 0.035). Patients with severe or critical disease were more likely to experience difficulty of breathing than those with mild or moderate illness (55.2% vs 44.8%, *p* < 0.001).

The median interval between the onset of symptoms and hospital admission was 5 days (IQR 3–8). The median duration of hospitalization for all patients was 8 days (IQR 6–12), longer for those with severe or critical disease than those with mild or moderate disease (11 days vs 8 days, *p* = 0.043). Almost half had at least one underlying disease (48.7%). Common co-morbidities were infectious diseases, including tuberculosis (17.4%), dengue (12.2%), and HIV (4.3%). Out of the 5 HIV patients, four had tuberculosis co-infection. Other infectious co-morbidities included syphilis (2.6%), diphtheria (1.7%), tetanus (1.7%), cerebral toxoplasmosis (0.9%), and rabies (0.9%). Of 115 patients, 21 (18.3%) had other illnesses aside from tuberculosis, dengue, and HIV. Non-infectious co-morbidities included type 2 diabetes mellitus (1.7%), Down syndrome (1.7%), bronchial asthma (0.9%), epilepsy (0.9%), major depressive disorder (0.9%), and obesity (0.9%). Having tuberculosis (*p* = 0.008) or at least one underlying disease (*p* < 0.001) was significantly associated with COVID-19 disease severity.

More than one third of all patients presented with pneumonia (38.3%). Patients with mild or moderate disease were more likely to present with an acute upper respiratory infection than those with severe or critical disease (95.5% vs 4.5%, *p* = 0.014). Two patients were diagnosed with acute respiratory distress syndrome, one of whom died. There were two reported cases of MIS-C, both in infants younger than 4 months old. Among all the cases, two individuals (1.7%) ages 13 and 14 years received 2 doses of BNT162b2 COVID-19 vaccine. The rest were unvaccinated (98.3%).

Ten children died (8.7%). Mortality was higher in those with severe or critical disease compared with mild or moderate disease (80% vs 20%, *p* < 0.001). Being diagnosed with sepsis or having at least one co-morbidity was associated with death, with *p*-values of 0.020 and 0.007 respectively (data not shown).

**Fig. 1:**
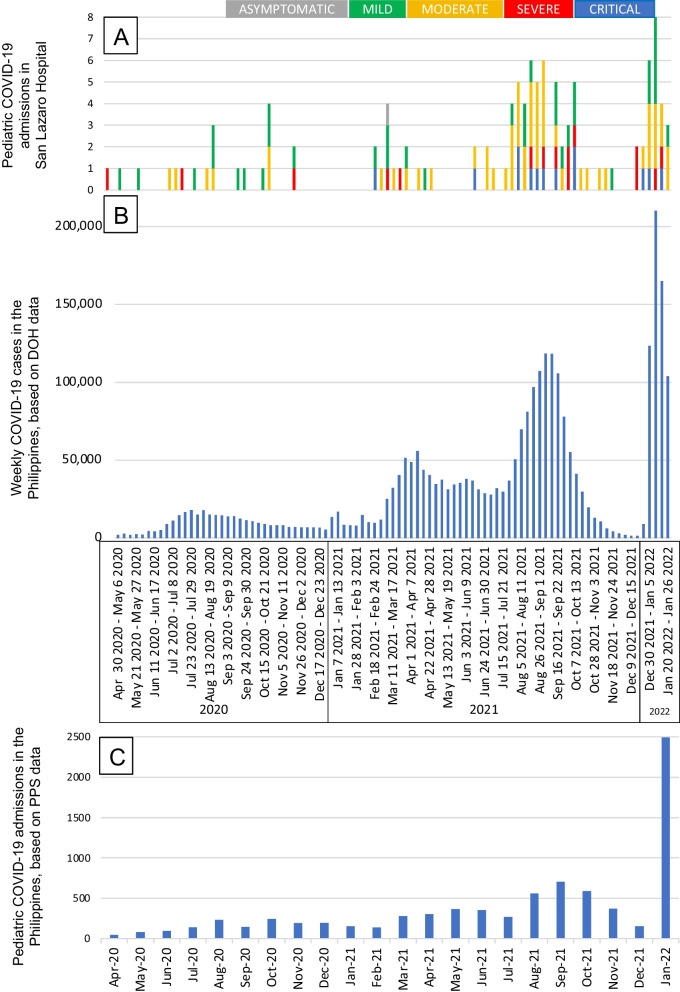
Distribution of confirmed COVID-19 cases between January 2020 and January 2022. **A** Pediatric COVID-19 admissions in San Lazaro Hospital, Manila, Philippines. **B** Total COVID-19 cases (all ages) in the Philippines per week, from DOH data. **C** Pediatric COVID-19 admissions in the Philippines, from PPS Registry data. COVID-19: coronavirus disease 2019; DOH: Department of Health; PPS: Philippine Pediatric Society

This study, which collected data within a 2-year period, encompassed admissions throughout the initial waves of the pandemic in the Philippines. As such, the circulation of the different variants may have affected the course and disease severity of the patients. The temporal distribution of confirmed COVID-19 cases is shown in Fig. 1. The trend of pediatric COVID-19 admissions in San Lazaro Hospital (Fig. 1A) resembled data on overall COVID-19 cases in the country (Fig. 1B); and paralleled that of hospitalized pediatric COVID-19 cases in the Philippines, based on a local disease registry program (Fig. 1C). As the number of cases surged in the country, so did the number of pediatric admissions. There was a notable uptick in pediatric COVID-19 admissions between July and October 2021, and another one beginning late December 2021, signifying circulation of the delta (B.1.617.2) and omicron (B.1.1.529) variants, respectively.

## Conclusion

As the COVID-19 pandemic continues, more people of all ages are getting infected and reinfected. Epidemiologic and clinical studies focused on children provide information on how SARS-CoV-2 affects the younger population, particularly in LMICs where reports are limited.

In this study, adolescents comprised a substantial proportion of the study population (40.9%), comparable to national data (35%) and an ongoing nationwide surveillance (32.2%) [19, 20]. COVID-19 admissions and mortalities in this study were mostly adolescents, highlighting the value of COVID-19 vaccination in this age group. The predominant clinical features of pediatric COVID-19 infection in this study mirrored that of other studies in children [21, 22]. Fever and cough were the most commonly observed symptoms, analogous to local reports [20, 23]. Six of the 10 patients who died had co-existing infectious diseases. The presence of concurrent infections may have adversely affected the COVID-19 illness of the patients, or vice versa. In a study in Brazil, the prevalence of co-morbidities was 50.6% in hospitalized pediatric COVID-19 patients who died [24].

This study has some limitations. The retrospective study design relied mainly on extraction of routinely collected data. Consequently, some expected variables were not available for inclusion in this study such as whether patients were diagnosed with COVID-19 prior to admission, or the proportion of patients referred from other hospitals or clinics. As this was a hospital-based research, the study population likely represented patients with more severe COVID-19 disease and hence, may not be reflective of the overall situation in the country. One patient with sepsis was classified as a moderate case, highlighting that assessment of COVID-19 disease severity may be challenging in cases where patients have other diagnoses. The limited sample size of this study may have precluded demonstrating other significant associations among variables. Further research involving a larger study population and multiple study sites may provide more details regarding this disease. Studies may be explored that focus on MIS-C in infants, co-infection of COVID-19 and other infectious diseases, COVID-19 and co-morbidities, and the effects of COVID-19 vaccination on disease severity in Filipino pediatric patients.

In conclusion, children of all ages remain susceptible to COVID-19 infection, and usually present with mild or moderate symptoms. Clinicians should be alert for children with difficulty of breathing as an indicator of severe or critical disease. Findings of this study provide insights into the clinical status of Filipino children with COVID-19 infection in a tertiary referral hospital in Manila, Philippines. Understanding the clinical features of this disease is essential to identify and manage children at highest risk of severe disease.

## Data Availability

The dataset for this study is available from the corresponding author and San Lazaro Hospital on a reasonable request. Data without names and identifiers will be made available after approval from the corresponding author and San Lazaro Hospital.

## Abbreviations

ACE2: Angiotensin converting enzyme II
CIF: Case investigation form
COVID-19: Coronavirus disease 2019
DOH: Department of Health
ED: Epidemiology Department
HIV: Human immunodeficiency virus
IQR: Interquartile range
LMIC: Low- and middle-income countries
MIS-C: Multisystem inflammatory syndrome in children
PPS: Philippine Pediatric Society
RT-PCR: Reverse transcription polymerase chain reaction
SD: Standard deviation
SLH: San Lazaro Hospital
SARS-CoV-2: Severe acute respiratory syndrome coronavirus–2
TB: Tuberculosis
WHO: World Health Organization
